# Implantation of a bone-anchored annular closure device in conjunction with tubular minimally invasive discectomy for lumbar disc herniation: a retrospective study

**DOI:** 10.1186/s12891-018-2178-4

**Published:** 2018-07-27

**Authors:** Frederic Martens, Geoffrey Lesage, Jeffrey M. Muir, Jonathan R. Stieber

**Affiliations:** 1 Department of Neurosurgery, OLV Ziekenhuis, Moorselbaan 164, 9300 Aalst, Belgium; 2 Motion Research, 3-35 Stone Church Rd., Suite 215, Hamilton, ON L9K 1S4 Canada; 3 Clinical Assistant Professor of Orthopaedic Surgery, New York University School of Medicine, 485 Madison Avenue, 8th Floor, New York, NY 10022 USA

**Keywords:** Annular closure device, Limited discectomy, Lumbar disc herniation, Microscopic discectomy, Minimally invasive, Tubular retractor

## Abstract

**Background:**

Minimally invasive techniques for lumbar discectomy have been recommended as superior to open techniques due to lower blood loss, lower rates of infection and shorter recovery. There are, however, concerns that this approach does not sufficiently remove the herniated nuclear material, thus leaving the patient susceptible to reherniation requiring reoperation. The purpose of this study was to examine the safety and viability of an annular closure device in limiting reherniation and reoperation in a cohort of patients undergoing minimally invasive lumbar discectomy with the assistance of an annular closure device.

**Methods:**

We retrospectively analysed the results from patients treated by a single surgeon between March 2011 and December 2017. All patients had been diagnosed with a large (≥ 5 mm) defect and were treated via minimally invasive surgical techniques. Outcomes included demographic data, the procedural duration and the rates of symptomatic reherniation and reoperation.

**Results:**

60 patients were included in the study. The mean age was 42 years (range: 19–66); mean BMI was 24.1 (range: 16.7–36.3). Mean surgical duration was 29 min (range: 16–50). Reoperation was required in 5% (3/60) of patients, although only 3% (2/60) experienced symptomatic reherniation at the index level. No other complications were reported.

**Conclusions:**

In our study, the use of an annular closure device during minimally invasive lumbar discectomy in a population of patients with large herniations was associated with low rates of reherniation and reoperation at the index level. While more research is required, the results of this study demonstrate the safety and viability of the annular closure device as an adjunct to minimally invasive discectomy.

## Background

Surgical discectomy has been proven as an effective treatment for lumbar intervertebral herniation; however, despite refinements of surgical approach and technique, there remains a persistent risk of recurrent reherniation at the index level [1–3]. Indeed, complications necessitating reoperation following discectomy occur in between 15 and 25% of cases [4–8].

Traditional discectomy techniques, first pioneered over 80 years ago, have been associated with generally good results but have also raised concerns regarding increased rates of surgical site infection [9, 10] and increased blood loss [11]. Such concerns have spurred the development of minimally invasive surgical (MIS) techniques that offer similar patient outcomes but without the added risks associated with traditional methods [11, 12]. Larger diameter tubes, bladed retractors, and advancements in technique have expanded the indications and complexity of minimally invasive spine surgery and have permitted the placement of biomechanical intervertebral devices for fusion purposes [13]. In limited discectomy, MIS techniques first centered around endoscopic access via a muscle-dilating, tubular approach. While more technically demanding than traditional open discectomy, the use of tubular discectomy spread more widely after the adaptation of the instrumentation and technique to direct visualization utilizing an operative microscope. While clinical outcomes have been shown to be equivalent between open and tubular techniques, there are concerns regarding reherniation rates, as minimal removal of nuclear material is thought by some authors to contribute to post-discectomy reherniation and, ultimately, reoperation [13, 14].

Maximizing clinical outcomes thus requires a balance between aggressive versus limited removal of nuclear material. On one hand, comparisons of limited versus traditional lumbar discectomy have demonstrated that while traditional discectomy, with its more extensive removal of disc material, substantially decreases the rate of recurrent disc herniation, aggressive nucleus removal leads to inferior clinical outcomes and patient satisfaction when compared with limited techniques, primarily due to disc collapse and subsequent back pain [15]. Conversely, other studies have demonstrated higher rates of reherniation in cases where limited techniques with minimal removal of disc material resulted in reherniation rates up to 27.3% of cases [15]. These observations were confirmed in a comprehensive review of the literature that found that limited techniques were associated with a higher rate of recurrent disc herniation but a lower rate of long-term recurrent back pain [16].

Ideally, the ability to combine the benefits of both traditional and limited techniques should be sought, to enable surgeons to routinely perform limited disc removal while also minimizing the risk of reherniation, a need that is underscored when considering patients with large annular defects (≥ 5 mm). As such, a novel annular closure device (ACD) has been developed to allow surgeons to retain maximal nuclear volume without increasing the risk of reherniation. The Barricaid® annular closure device has been in use for over a decade and has demonstrated an ability to decrease the rates of reherniation while allowing maximal preservation of nuclear volume at the time of surgery. While the early evidence from studies indicates that the use of this ACD results in a substantial reduction in reherniation rates [2], the use of this device with minimally invasive discectomy techniques remains uncharacterized. Proper placement of the ACD presents challenges unique to MIS techniques, as the facet joints must be preserved without predisposing injurious traction of the neural elements or incidental durotomy. To address these questions, we examined the feasibility, safety and risk of peri-operative complications of tubular minimally invasive insertion of the ACD as an adjunct to microdiscectomy with limited nucleus removal in a cohort of patients with large annular defects.

## Methods

### Study design

This study was a retrospective review of patients who underwent a lumbar discectomy procedure utilizing a limited surgical approach and an annular closure device. Ethics approval for this study was received from the participating institution. All participants provided informed consent prior to data collection.

### Patient eligibility

Patients were eligible for inclusion if they underwent a limited lumbar discectomy procedure between March 2011 and December 2017. Implantation of the ACD as an adjunct to limited tubular minimally-invasive lumbar discectomy was indicated in patients who met the following indications: 1) unilateral, single level lumbar disc herniation demonstrated on computed tomography and/or magnetic resonance imaging; 2) persistent radiculopathy and positive tension signs in both straight and crossed leg raising tests; 3) concordant radicular neurological deficits; and 4) intra-operative measurement of a large annular defect measuring 5–12 mm in width. Contraindications included posterior disc height ≤ 5 mm, spondylolisthesis greater than 25% (Grade II or higher) and osteoporosis.

### Annular closure device

**Fig. 1:**
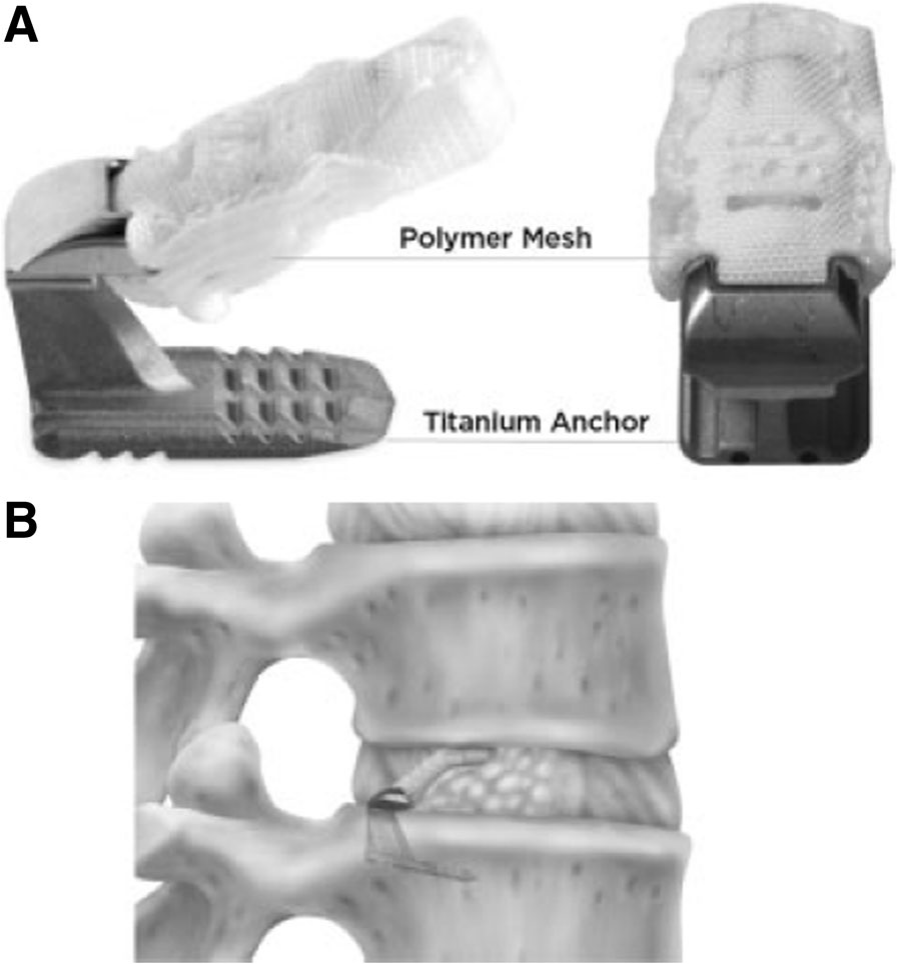
The Barricaid® implant showing sagittal (**a**) and posterior views and in the implantation site (**b**)

**Fig. 2:**
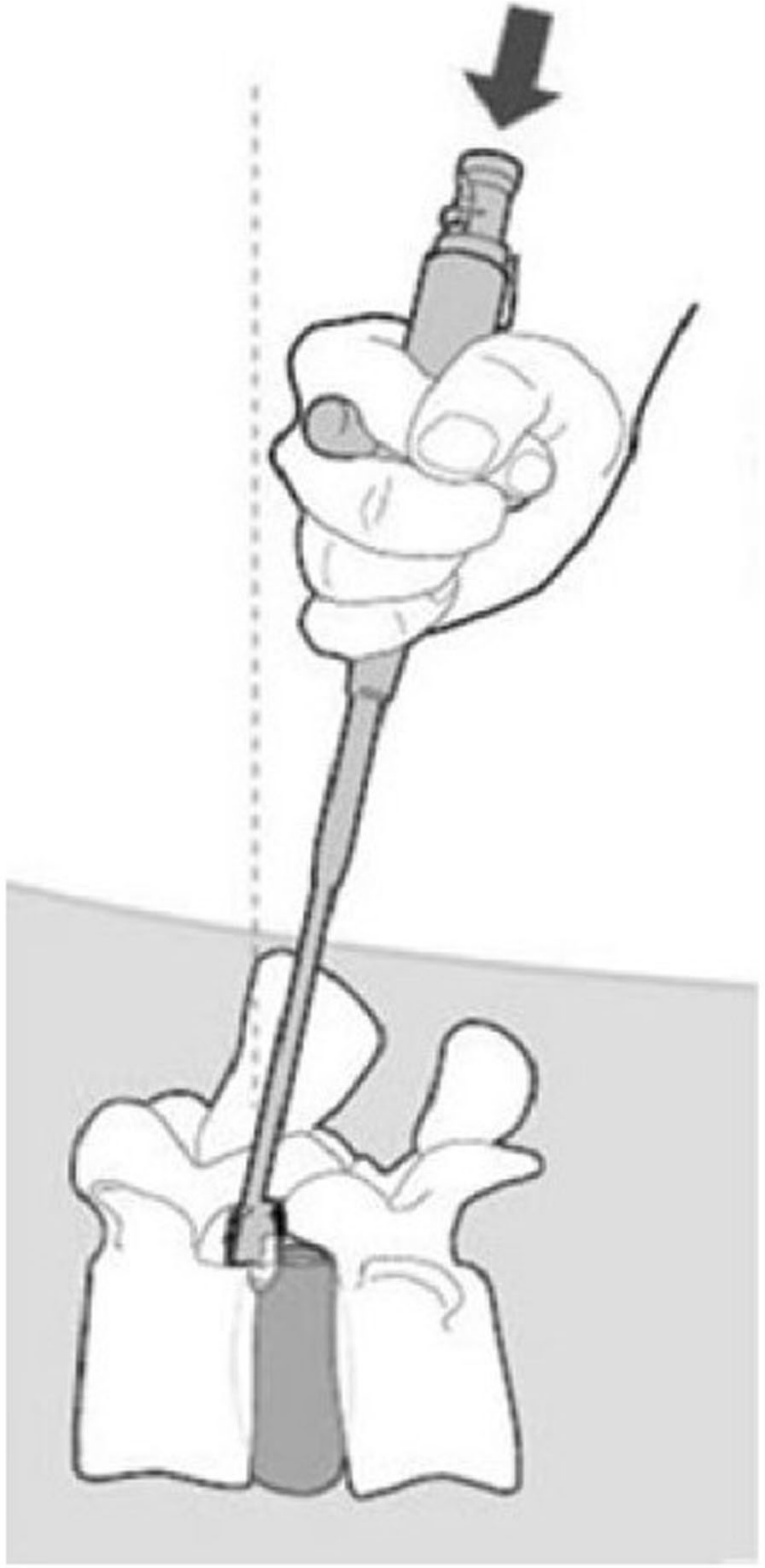
Schematic representation of Barricaid® endoprosthesis implanted in targeted disc space, by means of specialized delivery tool

The Barricaid® (Intrinsic Therapeutics, Inc., Woburn, MA, USA) is an annular closure device that has been CE-marked since 2009. The use of this ACD has been described in detail elsewhere [1, 2, 17]. In brief, the device is implanted following lumbar discectomy and is designed to retain nucleus pulposis within the disc space. The device consists of a flexible polymer (polyethylene terephthalate) occlusion component intended to block the opening in the annulus and prevent migration of the nucleus from within the disc, affixed to a titanium (Ti6Al4V ELI) bone-anchor that secures the occlusion component to one of the adjacent vertebral bodies (Fig. 1). A platinum iridium (radiopaque) marker on the occlusion component permits radiographic visualization and confirmation of its position. The device is available in 8-, 10-, and 12-mm widths and comes pre-loaded onto a disposable insertion tool (Fig. 2). The ACD is designed for herniations caused by extrusion of the nuclear material; however, as the device is anchored to the bony endplate, avulsion of the cartilaginous endplate material as part of the herniation [18] does not represent a contraindication. In such cases, the anchoring to the bony endplate is sufficient to secure the device in place and prevent further reherniation of the disc material.

### Surgical procedure

**Fig. 3:**
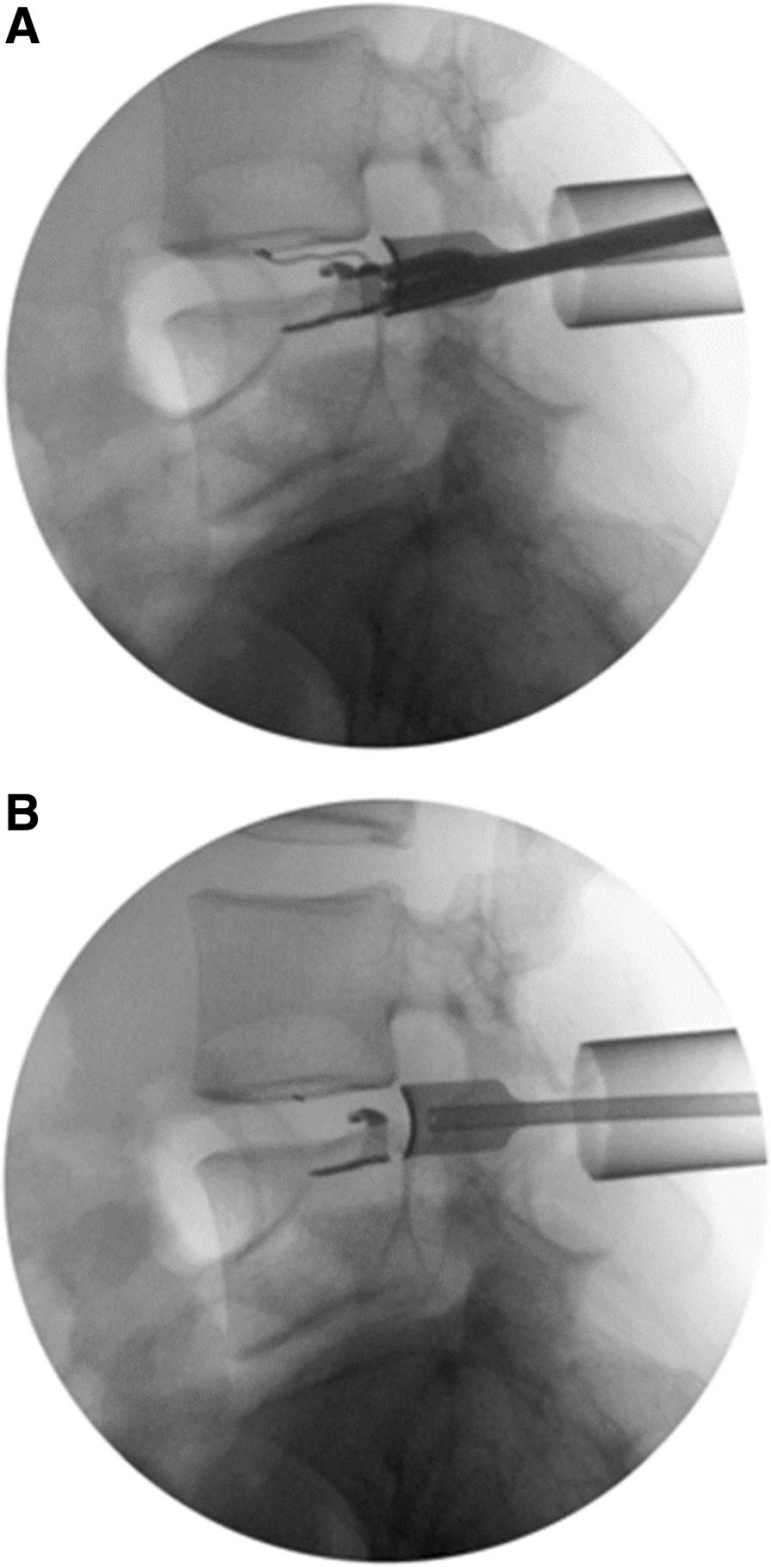
Fluoroscopic imaging demonstrating the Barricaid annular closure device during (**a**) and immediately following (**b**) implantation

Microdiscectomy was performed utilizing a 22 mm diameter fixed tube per standard technique using a tubular microdiscectomy system (METRx™ MicroDiscectomy System, Medtronic Memphis, TN, USA) in either a knee-on-chest position or utilizing a Wilson frame according to surgeon preference. Intraoperative fluoroscopy was utilized in order to obtain lateral images necessary for precise ACD sizing and implantation in addition to normal localization (Fig. 3). In order to facilitate proper ACD placement, the tube was aligned in the plane of the disc space. Visualization was achieved via an operative microscope. A limited discectomy was then performed, removing only extruded fragments and loose pieces of disc material within the disc space utilizing a pituitary rongeur.

**Fig. 4:**
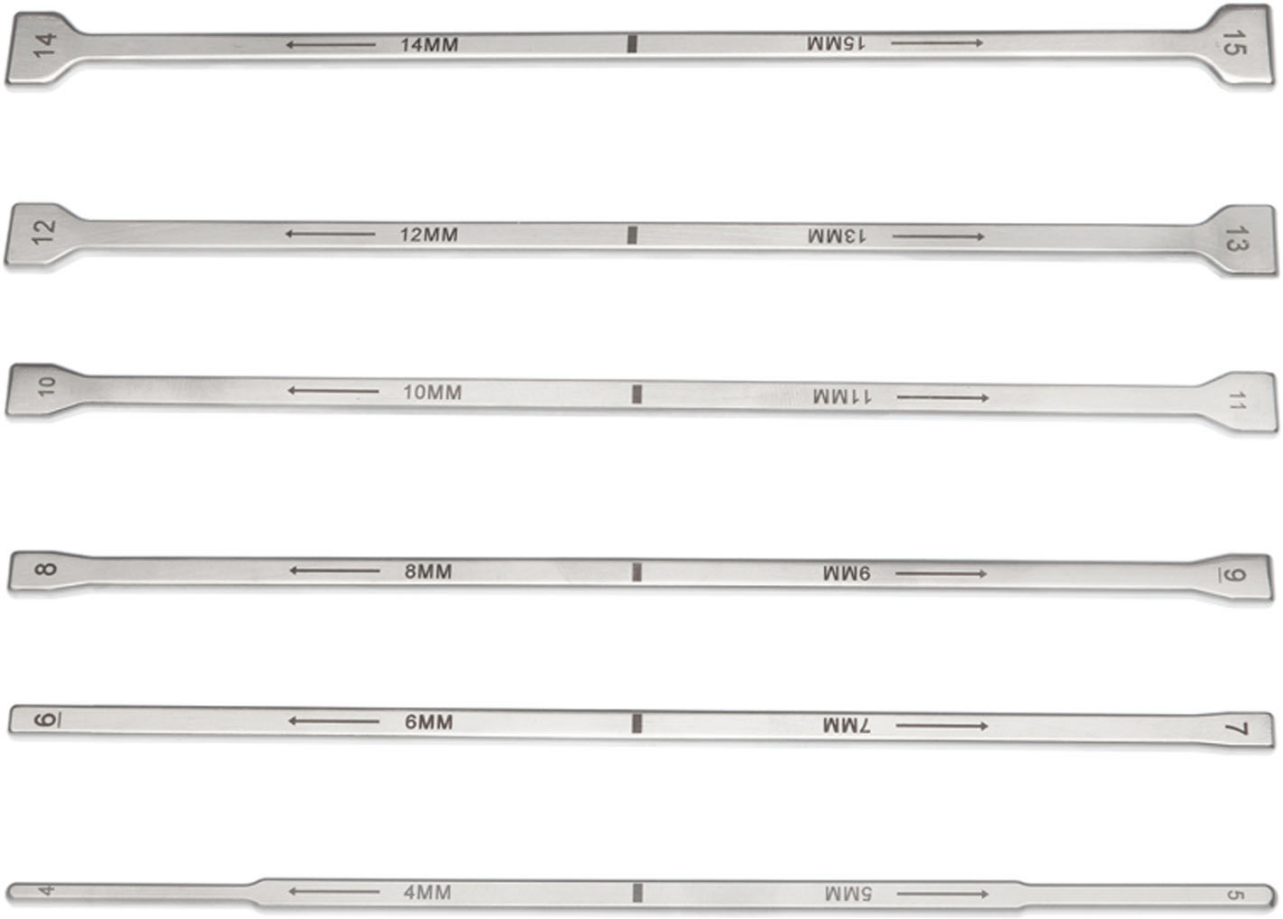
Eligibility for the annular closure device is determined using specific measurement tools to determine the defect size

**Fig. 5:**
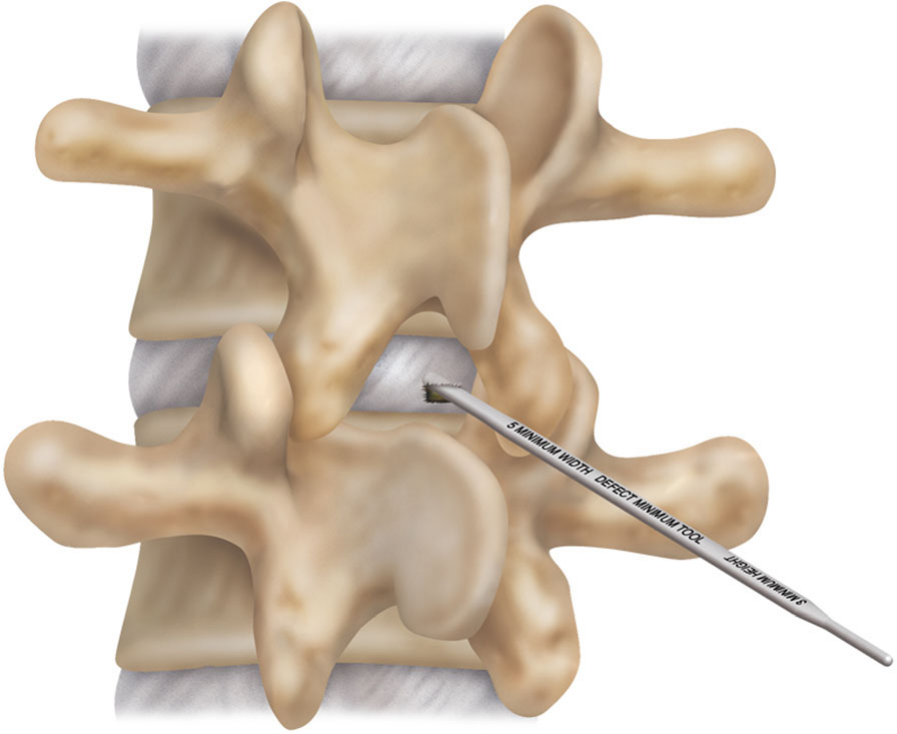
Schematic representation of defect size measurement

Following discectomy, the annular defect was assessed and measured with specifically-designed measuring tools. The height and width of the defect were measured by inserting dedicated defect measurement tools of varying sizes into the annular defect. Defect size was thus determined based on the best fit of the measurement tools (Figs. 4 and 5). In order to properly accommodate the ACD, the posterior disc height must measure at least 5 mm, per the Barricaid patient inclusion criteria and evidence in the literature that demonstrates the high incidence of symptomatic recurrent lumbar disc herniation in patients with large annular defects [15]. While there is no absolute limit on the height of the disc that can be implanted, the height of the annular defect should not exceed 6 mm. The width of the defect should not exceed the width of the mesh selected for insertion (8 mm, 10 mm or 12 mm).

Intraoperative data recorded during surgery included procedural duration and the volume of disc material retrieved. Patients were discharged with standard post-operative precautions. No additional bracing or non-standard activity restrictions were prescribed for any patient.

### Outcome measures

The primary outcomes for this study were the rate of symptomatic reherniation and reoperation at 6-month, 1-year and 2-year follow-up appointments. Symptomatic reherniation was defined as symptomatic sciatica with or without neurological deficit, with corroborating magnetic resonance (MR) imaging evidence. Secondary outcomes included operative time. Additionally, demographic data (gender, age, body mass index (BMI), surgical level) was also collected for each patient. Patients were followed up at serial timepoints up to 2-years and annually thereafter. Follow-up appointments included radiographic, MRI and CT imaging to confirm the integrity and positioning of the ACD.

### Statistical analysis

Alpha was set *a priori* at 0.05 for all statistical comparisons. Mean values were compared using independent samples t-tests, single-factor ANOVA or chi-squared tests, as appropriate. Mean values are expressed as mean (standard deviation).

## Results

### Study cohort demographics

A total of 60 patients were included in this study. The mean age of the participants was 42 years (range: 19–66); 58% (35/60) were female. The mean BMI was 24.1 (range: 16.7–36.3). All patients were diagnosed intraoperatively with large annular defects (≥5 mm) and met the indication for implantation of the ACD. Vertebral levels L4/L5 (38%, 23/60) and L5/S1 (52%, 37/60 were addressed surgically.

### Outcome measures

The mean operative time was 29 min (range: 16–50) from incision to wound closure. At 6-months post-procedure, no reoperations had occurred. At 1-year follow-up, symptomatic reherniation at the index level was reported in 3% (2/60) of patients, with reoperation at the index level likewise required in 2 patients (3%). In both cases, the reherniation occurred on the contralateral side of the implant. Reoperation at a level other than the index level was required in one additional patient, where herniation occurred on the contralateral side at a different level approximately 16 months following their initial discectomy.

At 2-years post-procedure, only 1 additional patient (2-year total: 3/60, 5%) required reoperation, although not at the index level or side. Three-year follow-up data was available for 29 patients and indicated that no additional reherniations or reoperations at any level were reported.

## Discussion

Despite advancements in the surgical technique, patients undergoing discectomy continue to be at risk for both recurrent disc herniation and subsequent disc degeneration with resulting back pain, both of which can compromise surgical outcomes and lead to revision surgical treatment. One method of minimizing the likelihood of reherniation is annular closure. One available device, the Barricaid ACD, is designed to maintain the favorable functional outcomes and patient satisfaction observed with limited discectomy, while also minimizing the risk of recurrent disc herniation in patients with large annular defects. We examined the rate of symptomatic reherniation and reoperation in a cohort of patients who underwent lumbar discectomy via a minimally-invasive approach and found that the use of the ACD was associated with lower rates of reherniation and reoperation when compared with rates observed elsewhere [15, 19].

The clinical advantages of tubular minimally invasive discectomy versus conventional discectomy have been examined in different clinical studies with the tubular approach found to be safe and effective [10, 20]. For both patients and surgeons, there continues to be widespread appeal for this approach. For MIS techniques combined with closure of the annulus, safe insertion of the device may be accomplished through the use of a 22 mm tube. Although implantation through smaller tubes is possible, the increased visibility obtained with the use of the customized combined suction-retractor facilitates safe passage of the ACD adjacent to the neural elements. The ACD can be appropriately placed without damage to the facet joint or sacrificing the stability of the posterior elements. While implantation of the ACD requires greater attention be paid to the location of the incision and the angle of approach than for traditional microdiscectomy, there is no increase in procedural time associated with the ACD and no increased risk of injury. Several studies have demonstrated the time-neutral nature of minimally-invasive discectomy [21, 22]. Indeed, the procedural time for this study (30 mins) was less than that measured in previous studies of minimally-invasive discectomy [22].

Symptomatic reherniation at the index level was observed in our study to have occurred in 2 of 60 patients (3%) by the 1-year mark, a rate that mirrors that of other studies of minimally-invasive discectomy but is significantly lower than that of other studies of large annular defects. In studies of limited discectomy for large annular defects, reherniation rates of up to 18% have been reported [15], suggesting that limited techniques may be lacking when used to treat large defects. Studies of MIS techniques, in contrast, have reported reherniation at rates similar to our study. A recent systematic review of MIS techniques for lumbar discectomy [23] found a pooled rate of reherniation of 2%, although the included studies did not focus on patients with large annular defects. That our study was able to demonstrate similar rates of reherniation in a smaller population of large annular defects is noteworthy, as is the long-term data available from our cohort. In our study, reherniation occurred within 1 year of the index procedure, with only one additional reoperation reported beyond 1-year. This finding is consistent with other studies of this ACD, which found significantly lower rates of reherniation and reoperation up to 2 years in ACD patients versus traditional discectomy [24]. In fact, our cohort was followed-up annually and in the 29 patients with a minimum of 3 years of follow-up, no subsequent instances of reherniation or reoperation were reported. That these patients reported no complications over that long period of time speaks to the value of the ACD in stabilizing the disc and preventing the herniation of the remaining disc material. The ability of the ACD to decrease reherniation rates has been demonstrated in other studies of this device [1, 2, 17, 19, 25] but the current study represents the first reported use of the Barricaid ACD with minimally-invasive techniques. As such, the potential for long-term stability following the use of this device warrants further and more rigorous study.

Our study demonstrated the excellent safety record associated with this device in this minimally-invasive setting, with no adverse events in 60 procedures, reflecting the overall safety of the procedure in general [20]. Previous studies using this ACD [17, 19, 26] have demonstrated similar results. MIS itself has been the subject of a comprehensive systematic review, evaluating the rate of complications in MIS lumbar discectomy versus open surgical methods [27]. These authors pooled results from 42 studies and found that MIS techniques were associated with lower rates of nerve root injury, wound complications and reoperation when compared with open techniques. MIS was also associated with decreases in length of stay, blood loss and the peri-operative risk of infection when compared with open techniques [12, 22]. The combined safety profiles of the ACD and MIS techniques – plus the current evidence of no adverse events when these techniques are combined – suggests that the use of the ACD with MIS techniques is a safe and effective method for addressing large lumbar annular defects.

Our study has limitations. Primarily, the observational nature of our study and the lack of a matched control group limit the veracity with which the results can be extrapolated. This study; however, successfully demonstrates the potential of the ACD during minimally-invasive lumbar discectomy, in a cohort of age-appropriate patients. Although more rigorous data collection in the form of a randomized, controlled trial in this cohort is required, these early results suggest a potential role for the ACD in minimally-invasive lumbar discectomy. There is also the potential for selection bias in this study, as the patients were all treated by the same surgeon and may represent a group of patients with an inherently greater likelihood of positive outcomes. However, the demographics of this cohort are reflective of the population of patients that would undergo such a procedure. As such, the likelihood of patients being selected for success is minimal, as the cohort represents one that would be operated on using these techniques under normal clinical circumstances.

## Conclusion

Our study demonstrated the viability of an annular closure device as an adjunct to minimally-invasive tubular lumbar discectomy in patients with large (≥ 5 mm) annular defects. We demonstrated decreased rates of symptomatic reherniation and reoperation in a representative cohort, when compared with known rates. While more rigorous study in higher level studies is required, the early results demonstrate the potential of the ACD in this operative setting.

## Abbreviations

ACD: Annular closure device
BMI: Body mass index
MIS: Minimally-invasive surgery
MR: Magnetic resonance
